# Ebola, Zika, Corona…What Is Next for Our World?

**DOI:** 10.3390/ijerph17093171

**Published:** 2020-05-02

**Authors:** Jagdish Khubchandani, Timothy R. Jordan, Y. Tony Yang

**Affiliations:** 1 College of Health, Ball State University, Muncie, IN 47306, USA; 2 College of Health and Human Services, University of Toledo, Toledo, OH 43606, USA; timothy.jordan2@utoledo.edu; 3 Center for Health Policy and Media Engagement, George Washington University, Washington, DC 20006, USA; ytyang@email.gwu.edu

**Keywords:** coronavirus, pandemics, global health, COVID-19, public health, poverty, population, pollution, globalization

## Abstract

In the past century, there have been several pandemics. Within the context of global health, these pandemics have often been viewed from the lens of determinants such as population, poverty, and pollution. With an ever-changing world and the COVID-19 pandemic, the current global determinants of public health need to be expanded. In this editorial, we explore and redefine the major determinants of global public health to prevent future pandemics. Policymakers and global leaders should keep at heart the determinants suggested hereby in any planning, implementation, and evaluation of efforts to improve global public health and prevent pandemics.

## 1. Background

In the 21st century, we have experienced infectious disease epidemics and pandemics and have heard/read about ones that may emerge in the future. The most recent experience for the world is the COVID-19 pandemic (Coronavirus Disease 2019). The epidemics that we have experienced in this century are not as new as many believe. Instead, the viruses that have caused these epidemics have been around for quite some time, continue to evolve, and in some cases, have worsened [1,2,3,4]. For example, Ebola viral infections first emerged in the 1970s, even though the current epidemics in Africa seem to be more troublesome. Similarly, the first human cases of Zika virus infections can be traced back to 1950s, with a major pandemic unfolding within the past few years. Coronaviruses are no strangers either. The first cases in humans were identified in 1960s with variants appearing as SARS (2003), MERS (2012), and the latest, COVID-19 [1,2,3,4].

In all the clamor and chaos surrounding the recent COVID-19 pandemic, we believe that the world leaders and public health experts have often failed to contextualize and clearly articulate the fundamental reasons *why* people across the world are experiencing an upsurge in novel, emerging, or reemerging infectious diseases. This failure in recognition and communication cuts across nations, systems, and organizations. We must clearly answer this question: Why are certain infectious diseases and epidemics on the rise, contrary to earlier predictions of global declines?

## 2. The Three Ps: Global Health Determinants

In 1992, Dr. Warren Winkelstein Jr., a celebrated epidemiologist at the University of California, wrote about the works of another highly decorated physician epidemiologist, Sir Richard Doll [5,6]. Professor Winkelstein categorized the work of Sir Doll into three major determinants of worldwide health: (1) Population, (2) Poverty and, (3) Pollution. These “3 Ps” are even more relevant today and should be used to examine the COVID-19 pandemic and to prevent future epidemics and pandemics [5,6,7].

The first “P” stands for population. Today, the world’s population is approximately 8 billion people. Projections indicate that by 2050, the world’s population could rise to 10 billion. Much of this population growth will be seen in less developed areas of the world. In addition, population growth worldwide will be accompanied by major demographic and epidemiologic transitions. For example, we will likely see more globalization and travel, refugees and migrants, warfare and conflict, exchange of goods and information, irresponsible consumption and unsustainable communities, cultural diffusion and social changes. These and other factors make current and future population growth unique and constitute a distinct syndrome (instead of mutually exclusive and separate set of changes) [7,8,9,10,11,12,13].

A salient example of these population factors is how human cases of COVID-19 in China triggered various changes in policies and practices around the world [13,14,15]. Given the potential for the spread of COVID-19, we saw worldwide changes in travel policies, trade policies, supply chains, and health care practices. Despite these changes, COVID-19 has spread to all countries in the world and is wreaking havoc in areas where the population density is greatest. Hence, starting now and into the future, public health specialists and medical experts must continue to further explore the association between demography and infectious disease. Family planning and gender equality should take a centerstage in global health security discussions, policies, and practices [8,9,10,11,12,13].

The second “P” stands for poverty. Everywhere around the world, poverty is a major determinant of health for individuals, communities, and nations. As of today, it is estimated that a tenth of the world’s population—750 million people—live on less than $2 per day. Around the world, poverty is associated with a multitude of risk factors for deleterious health outcomes such as: unemployment or insecure employment; homelessness and mental illness; drug use and violence; poor sanitation and unsafe drinking water; poor housing and dangerous living conditions; poor nutrition and unhealthy lifestyles; greater urbanization and unregulated industrialization; lower levels and poor quality of education; lower health awareness and lack of healthcare access. Poverty is strongly linked with the emergence and spread of existing and emerging infectious diseases [9,10,11,12,13,15,16,17,18].

The poor are disproportionately concentrated in developing countries of Asia, Africa, and the Americas. Therefore, it is no surprise that the recent epidemics and pandemics have originated in these parts of the world (e.g., 2013 Ebola virus epidemic in West Africa, 2015 Zika virus epidemic in Brazil, and COVID-19 epidemic in China). The COVID-19 pandemic will undoubtedly and exponentially increase the rate of poverty across the world. Especially at risk financially are those who were working as daily wage, part-time, or minimum wage workers. The alleviation of poverty is prominently featured among the UN Sustainable Develop Goals (goal number 1) [9,10,11,12,13,14,15,16,17,18]. However, greater efforts are needed to protect the most disadvantaged across the world from becoming a part of the vicious cycle of “poverty-infectious disease-greater poverty.”

The third “P” stands for pollution. Pollution is finally being recognized as a major determinant of health, or at least is now being talked about to a greater extent [19,20]. In regards to pollution, a spectrum of topics must be considered such as: climate change and extreme temperatures, land degradation and soil infertility, toxic chemicals and industrial discharge, floods and droughts, air pollution and respiratory disorders, ecosystems and agriculture, water quality and enteric disorders, environment and wildlife, waste generation and ocean acidification, deforestation and loss of biodiversity, drug resistance and germ mutations, greenhouse gases and ozone layer depletion, human-pathogen interactions, chronic diseases, and zoonosis [11,12,14,18,19].

The link between pollutant exposures among humans and infectious disease is well established . This link is of particular interest since many of the recent epidemics and pandemics such as Ebola, Zika, and COVID-19 have prominent respiratory modes of transmission. Furthermore, those with underlying respiratory diseases, some caused by exposure to air pollution, are at greater risk of serious illness and death. Climate action, clean energy, and clean water are three major goals in the list of UN Sustainable Develop Goals .Hence, starting now and into the future, global leaders should redouble their efforts to prevent and control pollution [9,10,11,12,13].

It is important to note that the “3 Ps” are interrelated and dynamically influence each other in a reciprocal relationship via multiple mechanisms. Hence, controlling one “P” may influence the other two in positive ways. Likewise, failing to account for or neglecting one “P” may negatively impact the other two. A prominent example of this reciprocal relationship can be seen in the 2003 H5N1 avian influenza outbreak in China [10,11,12,13,14]. A higher population density in low-and middle-income countries and larger family size were linked to poverty and greater demand for resources. The demand for employment, income, and food caused an intensification of animal husbandry practices without safety and sanitation. In those geographic areas with greater animal–human exposure and unregulated industrialization, H5N1 infections were facilitated by these factors and by weak regulations. The transmission of avian influenza H5N1 in China has been frequently explained via this interrelatedness of population, poverty, and poultry production [11,12,13,14,18,19,20].

## 3. COVID-19 Pandemic: Need for Expansion of the Three Ps

Given the recent epidemics and pandemics, the ideas of Sir Hill and Professor Winkelstein are still very relevant [5,6,7,8]. However, the world has seen many changes since the “3 Ps” concept was originally proposed. Therefore, we believe that an expansion of the “3 Ps” model is warranted to better understand recent pandemics, explore additional determinants of global public health, and to create better plans and prevention strategies for the future. We recommend that the additional “Ps” delineated below be added to the original model.

### 3.1. Publicity and Panic

Since the “3 Ps” were proposed, there have been significant changes and major proliferations in mass media, social media, and Internet-based communication. Such methods and modes of information sharing and communication are now a major influence on how people understand health, its determinants, and how they respond to health crises. Hence, we recommend that a fourth “P” be added to the original model: “Publicity and Panic.”

Mass and social media, technology and communication, propagation of misinformation and myths, conspiracy theories and rumors have become major drivers of anxiety and panic during the COVID-19 pandemic. During the current crisis, the world is fighting an “infodemic” along with a pandemic. Such an “infodemic” points out the need for a comprehensive, coherent, and sensible approach for a global flow of valid and reliable scientific information. Providing people of all nations with accurate and truthful public health and medical information will help create greater awareness and trust while simultaneously reducing the burden of misinformation and panic that may accompany global pandemics. Unfortunately, working to improve this determinant of global public health will be difficult because social and mass media are controlled, utilized, and protected differently around the world with an added burden of lack of free press in many regions [20,21,22,23].

In response to pandemics like COVID-19, technological approaches to improving public health awareness, medical surveillance, artificial intelligence, big data generation, and telemedicine should be researched further to enhance their efficacy, reach, acceptability, and affordability [22,23]. Of particular concern are the poor and marginalized populations around the world who have lower levels of education and suffer the most from a global digital divide. Such populations typically do not have wide access to mass media, social media, and Internet access. As we discussed previously, such populations are also the most negatively and profoundly impacted by infectious disease outbreaks. Hence, special attention, comprehensive outreach, and low-cost means will be needed to help these populations access valid and reliable sources of health-related information and digital healthcare services [11,18,20,21,22,23].

### 3.2. Politics and Policies

Another new “P” that we recommend be added to the original model is “Politics and Policies.” Health is profoundly political and global health is significantly influenced by politics. The decisions of the world’s political leaders determine policies, priorities, practices, and funding for public health and health-related research. Furthermore, political decisions and their associated policies greatly influence what is studied or researched, environmental regulations, public-private partnerships, and social processes and institutions. One can easily see how politics and policies are dominant influences on global health [24,25,26].

Unfortunately, the response to the COVID-19 pandemic has been mired by politics within and among countries. The pandemic arrived at a critical time when the world was afflicted by trade wars and protectionist practices, socioeconomic and health disparities among countries, decreased funding for public health and health related research, geopolitical distrust and lack of transparency, shrinking support for global health initiatives and development, bioterrorism and nuclear threats, differing views and actions of nations on global health governance and security, microeconomic instability and macroeconomic fragility (that will be accelerated further by the COVID-19 pandemic) [7,8,14,15,18,20]. These factors contributed a to the lack of a timely, unified, coordinated, and comprehensive international strategy in response to the COVID-19 pandemic.

It remains to be seen how global political leaders and organizations deal with the pandemic in the short term and effectively address the original “3 Ps” in the long run (i.e., pollution reduction, poverty alleviation, and population control). Considering the current chaotic, distrustful, and antagonistic political climate that we see today, we recommend that global leaders redouble the efforts and their commitment to a better world as highlighted in the UN Sustainable Goals (especially Goal Number 17: Revitalize the global partnership for sustainable development) [9,10,11]. Without trust, cooperation, compromise, accountability, and collaboration within and among nations, the consequences of the current pandemic (and future ones) will only be more severe.

### 3.3. Preparedness and Products

A fundamental challenge to effective responses to pandemics such as COVID-19 lies in the degree of preparedness of public health and healthcare. Preparedness also includes the availability of medical personnel, products, and physical structures (e.g., hospital and community health workers, personal protective equipment and sanitizers, reagents and swabs, ventilators and beds, medications and vaccines). Thus, we recommend adding a sixth “P” to the original model: “Preparedness and Products.”

Given the recent history of pandemics, “preparedness” needs to be redefined. The changing world today demands that preparedness not be a static concept or a binary concept (prepared or unprepared), when most of the time we are “underprepared.” Instead, it should reflect a dynamic state within and among countries where harm can be reduced during pandemics such as COVID-19. In addition, we believe that preparedness is deeply intertwined with the other “Ps” [15,16,17,18,19,20]. For example, let us consider a large group of people living close together (i.e., high population density) in a slum in a large city in a developing country. They face a very high rate of poverty, unemployment, food insecurity, lack of sanitation and clean water. This population generally does not have access to formal health care or money to pay for high tech medical care in an intensive care unit. Due to their political leaders’ failure to address climate change or their government’s lack of economic resources to do so, this population will likely face increased heat, drought, or flooding, increased displacement, and increased exposure to various types of pollution. As this very poor population faces increasingly greater risks, what is likely to happen? It is easy to see how the interrelatedness of a multitude of risk factors spells doom for the preparedness of this population for future epidemics or pandemics.

The stressors, disruptors, and barriers to global and national preparedness for pandemics such as COVID-19 need further study and exploration. Future research might examine topics such as the changing biology and pathogenesis of disease agents, socioeconomic and environmental influences on preparedness, and the influence of political and institutional factors of preparedness [9,10,11,12,13,14]. Such investigations will be undertaken by the global scientific community and the fear is not about inadequate research, but lack of adequate action from the global political community (another obvious example of interdependence of “Ps”).

### 3.4. Primary Care

Healthcare is critical to human beings. Accessible and high-quality primary care is an essential component of any national or regional healthcare system that aspires to be effective, efficient, and equitable. Primary care systems are also the foundation and key drivers of pandemic related emergency response and can serve as warning barometers for emergency response mechanisms [24,25,26,27]. Therefore, the last “P” that we recommend adding to the original model is: “Primary Care”

The role of primary care is especially critical during and after pandemics. For example, during the early stages of the COVID-19 pandemic, 1751 primary care practitioners in China were asked about their practices and how they were dealing with the pandemic [28]. Almost half reported that they did not follow-up on suspected cases or failed to refer suspected cases for further evaluation, diagnosis, and specialty care. Evidently, neither systems nor practitioners, were responsive enough for a multitude of reasons [24,25,26,27,28,29,30]. It is interesting to note that Professor Warren Winkelstein, who we mentioned earlier, lamented the fact that students training for future professions in health care receive training that is too narrow. He went on to say that many health care providers tend to remain narrow in their profession and focus on a single or a very limited range of diseases over the course of a professional lifetime [5]. Such a narrow approach may contribute to deeper levels of knowledge and expertise in one area but limits health care providers’ knowledge of the overall health care system and public health. His comments point to the need to teach future health care providers how to address the health needs of populations as well as individuals [5,27,28].

Primary care practices see many more patients during seasonal illnesses (e.g., influenza). However, some researchers estimate that primary care health providers will experience an additional 1500 to 3000 primary care visits per 100,000 people during peaks of pandemics [27]. The COVID-19 pandemic may have created an even a higher burden. Are primary care health providers ready for this tidal wave? Have they been properly trained to handle the unique challenges of this pandemic? Do they have the appropriate personal protective equipment to stay safe? Do they have adequate training, technical equipment, and the ability to do effective telemedicine? These are all good questions that we will only be able to answer in the future.

The areas of concern for primary care in dealing with pandemics such as COVID-19 are many and remain ubiquitous across nations and regions [24,25,26,27,28,29,30]. These concerns include the lack of personal protective and basic medical care equipment; limited financial reserves and clinical capacity; rural-urban and geographic disparities; patients and practices with limited access to reliable and high speed Internet; fractured connections between public health services and emergency care; patient level factors such as inability to pay and low literacy; medical mistrust and treatment adherence issues; shrinking physician interest in general practice and growing demand for specialists; lack of emphasis on preventive screenings and vaccination; absence of interdisciplinary and integrated functions and systems; disparities in investment between horizontal and vertical programs; poor infrastructure and human resource depletion; inadequate compensation and low competence of professionals; absence of benchmarks and continuing education; lack of care coordination and information flow; issues of ethics and professionalism among providers; diverse models of care and compensation; functional weaknesses and poor social participation; conflict between community level gatekeeping and centralized care; lack of administrative support and leadership in primary care; lack of regulations for providers and paying mechanisms; and public versus private approaches to primary care [20,21,22,23,24,25,26,27,28,29,30]. Global political leaders and public health experts will need to collaborate and strategize to address each of these important issues with a multipronged approach, keeping in view the other “Ps”.

## 4. Looking Forward: During and After COVID-19 Pandemic

Given our proposed expansion to the global determinants of public health (Ps) and the unique nature and extent of trauma inflicted by COVID-19 on the modern world, there are many opportunities and challenges ahead. The issues related to the current pandemic are analogous to an iceberg. On the surface of the water, the tip of the iceberg is clearly visible. At the tip of the iceberg, we clearly see two things: (1) the lack of a coordinated, collaborative, comprehensive, and effective global response to the pandemic and (2) the human tolls of morbidity, mortality, grief, and increased unemployment and poverty. However, beneath the surface of the water lie the “causes of the causes”— the deeper, multiple determinants of global public health that will require renewed attention and sustained efforts from the global community.

As we move forward, there will be numerous silver linings, rays of hope, multiple calls for action, intellectual debates, post-mortem analyses, and plans and strategies within and among nations. However, the most critical need now is for the development and strengthening of intersectoral approaches and cross-nation collaborations to address all the “Ps”. One simple strategy would be for regional and global leaders to revisit the United Nations Sustainable Development Goals to be achieved by 2030 [9,10]. A fitting response to the current pandemic would be for global leaders to cooperate and collaborate on redoubling the efforts to collectively accelerate the achievement of the 17 global goals for human development.

No matter the way forward, it is very important and necessary that global leaders use a retrospective lens to reconsider past lessons from SARS, Ebola, and Zika epidemics. It is only through such retrospection that we will be able to forge a successful path forward. It is also vital that lessons from the current pandemic are learned. Finally, we strongly recommend that global and regional leaders consider, account for, and include our expanded global determinants of public health (“Ps”). Any way forward must include all of these primary determinants of global public health.

## References

[1] Kahn J.S., McIntosh K. (2005). History and recent advances in coronavirus discovery. Pediatr. Infect. Dis. J..

[2] Weyer J., Grobbelaar A., Blumberg L. (2015). Ebola virus disease: History, epidemiology and outbreaks. Curr. Infect. Dis. Rep..

[3] Weaver S.C., Costa F., Garcia-Blanco M.A., Ko A.I., Ribeiro G.S., Saade G., Shi P.Y., Vasilakis N. (2016). Zika virus: History, emergence, biology, and prospects for control. Antivir. Res..

[4] Ye Z.W., Yuan S., Yuen K.S., Fung S.Y., Chan C.P., Jin D.Y. (2020). Zoonotic origins of human coronaviruses. Int. J. Biol. Sci..

[5] Pincock S. (2012). Warren Winkelstein Jr. Lancet.

[6] Winkelstein W. (1992). Determinants of worldwide health. Am. J. Public Health.

[7] Khubchandani J., Simmons R. (2012). Going global: Building a foundation for global health promotion research to practice. Health Promot. Pract..

[8] Ubokudom S.E., Khubchandani J. (2010). The ecology of health policymaking and reform in the USA. World Med. Health Policy.

[9] United Nations Sustainable Development Goals. https://www.un.org/sustainabledevelopment/sustainable-development-goals/.

[10] Waage J., Yap C., Bell S., Levy C., Mace G., Pegram T., Unterhalter E., Dasandi N., Hudson D., Kock R. (2015). Governing the UN Sustainable Development Goals: Interactions, infrastructures, and institutions. Lancet Glob. Health.

[11] Merson M.H., Black R.E., Mills A.J. (2018). Global Health: Diseases, Programs, Systems, and Policies.

[12] McMichael A.J. (2013). Globalization, climate change, and human health. N. Engl. J. Med..

[13] Abel G.J., Barakat B., Samir K.C., Lutz W. (2016). Meeting the Sustainable Development Goals leads to lower world population growth. Proc. Natl. Acad. Sci. USA.

[14] Phelan A.L., Katz R., Gostin L.O. (2020). The novel coronavirus originating in Wuhan, China: Challenges for global health governance. JAMA.

[15] Barua S. (2020). Understanding Coronanomics: The Economic Implications of the Coronavirus (COVID-19) Pandemic (No. 99693).

[16] Fouilleux E., Bricas N., Alpha A. (2017). ‘Feeding 9 billion people’: Global food security debates and the productionist trap. J. Eur. Public Policy.

[17] Sharma P., Dwivedi S., Singh D. (2016). Global poverty, hunger, and malnutrition: A situational analysis. Biofortification of Food Crops.

[18] Heymann D.L., Chen L., Takemi K., Fidler D.P., Tappero J.W., Thomas M.J., Kenyon T.A., Frieden T.R., MBChB D.Y., Nishtar S. (2015). Global health security: The wider lessons from the west African Ebola virus disease epidemic. Lancet.

[19] Landrigan P.J., Fuller R., Acosta N.J., Adeyi O., Arnold R., Baldé A.B., Bertollini R., Bose-O’Reilly S., Boufford J.I., Breysse P.N. (2018). The Lancet Commission on pollution and health. Lancet.

[20] Madhav N., Oppenheim B., Gallivan M., Mulembakani P., Rubin E., Wolfe N. (2017). Pandemics: Risks, impacts, and mitigation. Disease Control Priorities: Improving Health and Reducing Poverty.

[21] Garfin D.R., Silver R.C., Holman E.A. (2020). The novel coronavirus (COVID-2019) outbreak: Amplification of public health consequences by media exposure. Health Psychol..

[22] Xie B., He D., Mercer T., Wang Y., Wu D., Fleischmann K.R., Zhang Y., Yoder L.H., Stephens K.K., Mackert M. (2020). Global health crises are also information crises: A call to action. J. Assoc. Inf. Sci. Technol..

[23] Merchant R.M., Lurie N. (2020). Social Media and emergency preparedness in response to novel Coronavirus. JAMA.

[24] Moon S., Sridhar D., Pate M.A., Jha A.K., Clinton C., Delaunay S., Edwin V., Fallah M., Fidler P.D.P., Garrett L. (2015). Will Ebola change the game? Ten essential reforms before the next pandemic. The report of the Harvard-LSHTM Independent Panel on the Global Response to Ebola. Lancet.

[25] Elbe S. (2018). Pandemics, Pills, and Politics: Governing Global Health Security.

[26] Holmberg M., Lundgren B. (2018). Framing post-pandemic preparedness: Comparing eight European plans. Glob. Public Health.

[27] Rust G., Melbourne M., Truman B.I., Daniels E., Fry-Johnson Y., Curtin T. (2009). Role of the primary care safety net in pandemic influenza. Am. J. Public Health.

[28] Xu Z., Qian Y., Fang L., Yao M. (2020). Primary Care Practitioners' Response to 2019 Novel Coronavirus Outbreak in China. medRxiv.

[29] Osterholm M.T. (2005). Preparing for the next pandemic. N. Engl. J. Med..

[30] National Academy of Medicine (2016). The Neglected Dimension of Global Security: A Framework to Counter Infectious Disease Crises.

